# Development of Loop-Mediated Isothermal Amplification (LAMP) assay for rapid detection of *Fusarium oxysporum* f. sp. *ciceris* - wilt pathogen of chickpea

**DOI:** 10.1186/s13104-015-0997-z

**Published:** 2015-02-11

**Authors:** Raju Ghosh, Avuthu Nagavardhini, Anindita Sengupta, Mamta Sharma

**Affiliations:** International Crops Research Institute for the Semi-Arid Tropics (ICRISAT), Patancheru, PO 502324, Andhra Pradesh, India

**Keywords:** Detection, *Fusarium oxysporum*, Hydroxynaphthol blue, Isothermal amplification, LAMP

## Abstract

**Background:**

*Fusarium oxysporum* f. sp. *ciceris* (Foc), the causal agent of Fusarium wilt is a devastating pathogen of chickpea. In chickpea, various soil borne pathogens produce (s) similar symptoms, therefore cannot be distinguished easily at field level. There is real need for a rapid, inexpensive, and easy to operate and maintain genotyping tool to facilitate accurate disease diagnosis and surveillance for better management of Fusarium wilt outbreaks.

**Results:**

In this study, we developed a loop-mediated isothermal amplification (LAMP) assay targeting the elongation factor 1 alpha gene sequence for visual detection of Foc. The LAMP reaction was optimal at 63°C for 60 min. When hydroxynaphthol blue (HNB) was added before amplification, samples with Foc DNA developed a characteristic sky blue colour but those without DNA or with the DNA of six other plant pathogenic fungi did not. Results obtained with LAMP and HNB were confirmed when LAMP products were subjected to gel electrophoresis. The detection limit of this LAMP assay for Foc was 10 fg of genomic DNA per reaction, while that of conventional PCR was 100 pg.

**Conclusions:**

In conclusion, it was found that a LAMP assay combined with HNB is simple, rapid, sensitive, and specific. The LAMP assay does not require specialized equipment, hence can be used in the field for the rapid detection of Foc. This is the first report of the use of LAMP assay for the detection of Foc. The presented LAMP method provides a specific, sensitive and rapid diagnostic tool for the distinction of Foc, with the potential to be standardized as a detection method for Foc in endemic areas and will be very useful for monitoring the disease complex in the field further suggesting the management strategies.

**Electronic supplementary material:**

The online version of this article (doi:10.1186/s13104-015-0997-z) contains supplementary material, which is available to authorized users.

## Background

Chickpea (*Cicer arietinum* L.) is the second largest cultivated legume crop after dry beans globally [1]. It is grown in 54 countries as a rainfed, post-rainy season and winter crop in subtropical South Asia, parts of Africa and Australia and as a spring season crop in the temperate and Mediterranean regions [1]. India is the largest producer of chickpea and accounts for 68.47% of the total area and 67.02% of total production globally. Chickpea represents 35.16% of total pulse area and 50.34% of total pulse production in India [2].

Various biotic and abiotic stresses affect stable and high yields of chickpea crop worldwide. Among the biotic stresses, Fusarium wilt (FW), caused by the asexual, soil borne and seed borne fungus *Fusarium oxysporum* Schlecht and Emnd Snyd. & Hans. f. sp. *ciceris* (Padwick) Snyd. and Hans. (Foc), results in major economic losses ranging from 10-40% worldwide. It is estimated to cause 10-15% yield loss annually in India [3], but can result in 100% losses under favourable conditions. The cultivation of resistant varieties is one of the most durable and economical practices for the management of FW. However, performance of varieties differs from place to place owing to existence of physiological races among the Foc isolates. Eight races of Foc (0, 1A, 1B/C, 2, 3, 4, 5 and 6) have been reported worldwide [4-6]. Races 1A (also known as race 1), 2, 3 and 4 have been reported from India, whereas races 0, 1B/C, 5 and 6 were found mainly in the Mediterranean region and in the United States (California). Recently, change in the distribution of Foc has been reported by Sharma et al. in India [7]. Therefore, to prevent the introduction and spread of Foc races in different new regions of India, a suitable, reliable and rapid detection method is prerequisite. In recent years, PCR-based methods, for instance multiplex and real-time PCR have been developed to detect fungal species and other microorganisms [8-11], however, methods based on PCR can be time-consuming and require the extraction of high-quality DNA due to the effects of inhibitors on PCR sensitivity [12,13]. Loop-mediated isothermal amplification (LAMP) is an alternative amplification technology [14], is highly sensitive, less time-consuming than conventional PCR-based methods, and less prone to inhibition from DNA preparations [15-18]. Amplification by LAMP involves the use of six primers (two internal, two external and two loop primers) and relies on auto cycling DNA synthesis by a DNA polymerase with high strand displacement activity. Both the forward and backward inner primers contain two distinct sequences each, corresponding to the sense and the antisense sequences of the target DNA. Amplification products are characterized by the fact that they contain loop regions to which further primers can bind, allowing the amplification to continue isothermally [14]. The speed of the reaction is accelerated using additional loop primers that bind to those loops which are of the inverse orientation to the loops to which the internal primers bind [19]. Like PCR, LAMP reaction can be monitored in real time using intercalating fluorescent dyes such as ethidium bromide, SYBR Green I, propidium iodide, or Quant-iT PicoGreen; by adding metal-ion indicators such as hydroxynaphthol blue (HNB) [20], CuSO_4_ [21], or calcein [17] or by measuring the increase in turbidity derived from magnesium pyrophosphate formation (to infer increases in amplified DNA concentration). LAMP products can also be detected by real-time detection methods [22].

The simplicity of the LAMP method, which does not require a thermal cycler, makes it suitable for field testing also. Recently, LAMP assay has been developed for the detection of bacteria [23], viruse [24], and fungi [25]. In chickpea, various pathogens exist simultaneously and difficult to distinguish visibly by looking at symptoms. Fusarium wilt is easily mistaken from Dry root rot, as the general symptoms of these diseases are similar. Affected plants show foliar chlorosis and causes mortality of the plants usually in patches in field. This presents a real need for a rapid, inexpensive and easy to handle tool to facilitate accurate disease diagnosis and surveillance for better management of Fusarium wilt. The LAMP method has been applied first time for the detection of Foc.

The purpose of the present study was to develop LAMP assay for the detection of Foc and to evaluate the diagnostic sensitivity and specificity of this assay using a panel of fungal DNA samples and infected field samples of chickpea. The newly developed LAMP assay successfully detected Foc with rapidity, specificity, and high sensitivity.

## Results

### Design of LAMP primers and assay

**Figure 1:**
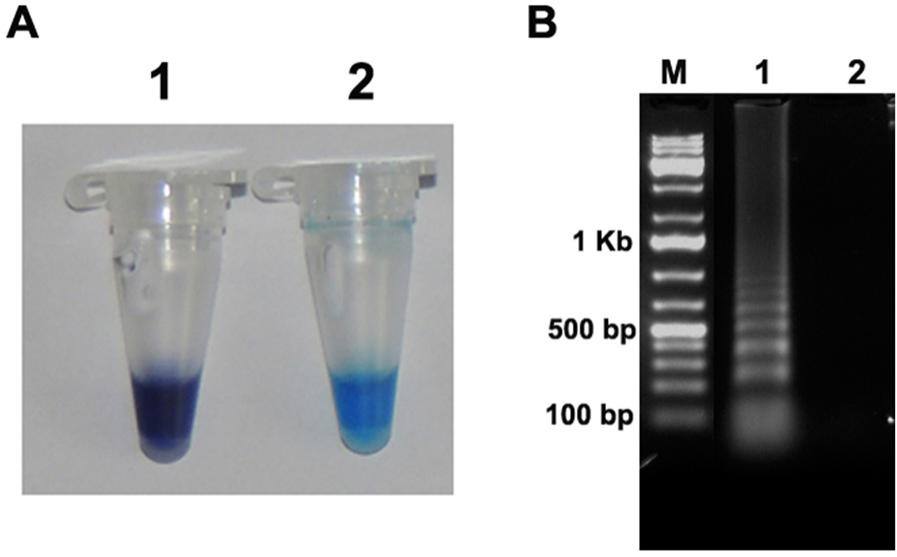
**Detection of elongation factor 1 alpha gene of** ***Fusarium oxysporum*** **f. sp.** ***ciceris*** **by LAMP. (A)** LAMP detection using HNB as a visual indicator. The reaction becomes sky blue if the target gene is present (Tube 2) but remains violet if the gene is absent (Tube 1). **(B)** Agarose gel electrophoresis of LAMP products. The positive reaction is manifested as a ladder-like pattern on the 1.5% agarose gel. M = 100 bp DNA marker. Lane1: Positive reaction (with target DNA). Lane B: Control (without template DNA).

For primer designing, various Foc isolates were examined to identify the conserved regions of the fungus genome and EF-1 alpha gene was chosen. A set of primers were designed by LAMP designer software based upon the conserved regions among isolates and used subsequently for the specificity of the LAMP assay. All the primers were tested by *in-silico* using the nucleotide BLAST search tool on the NCBI sequence database that revealed significant hits for the target sequences. When the LAMP assay was performed with Foc DNA as the template, the best results were obtained in a 25 μL volume containing 2.0-μl primer mixture (20 μM each of FIP, BIP, Loop F, and Loop B primers, and 2.5 μM each of F3 and B3 primers) 1.28 M betaine, 1 mM dNTPs, 4 mM MgCl_2_, 20 mM Tris–HCl (pH 8.8), 10 mM KCl, 10 mM (NH_4_)_2_SO_4_, 2 mM MgSO_4_, 0.1% Triton X-100, 8 U of *Bst* DNA polymerase, 150 μM HNB, and 1 μL of target DNA. As noted in the methods, the reactions were performed in a 0.2-mL microcentrifuge in a water bath for temperature control. When the tubes were examined before gel electrophoresis, a positive LAMP reaction was indicated by a sky blue colour; the colour remained violet for negative reactions (Figure 1A). After the tubes were visually assessed for colour change, the samples were subjected to agarose gel electrophoresis; characteristic bands were evident in the gel if the product was present but not if the product was absent (Figure 1B). The results showed that the primers were effective, and that the same result was obtained with HNB visualization and gel electrophoresis.

### Optimization of LAMP reaction conditions

**Figure 2:**
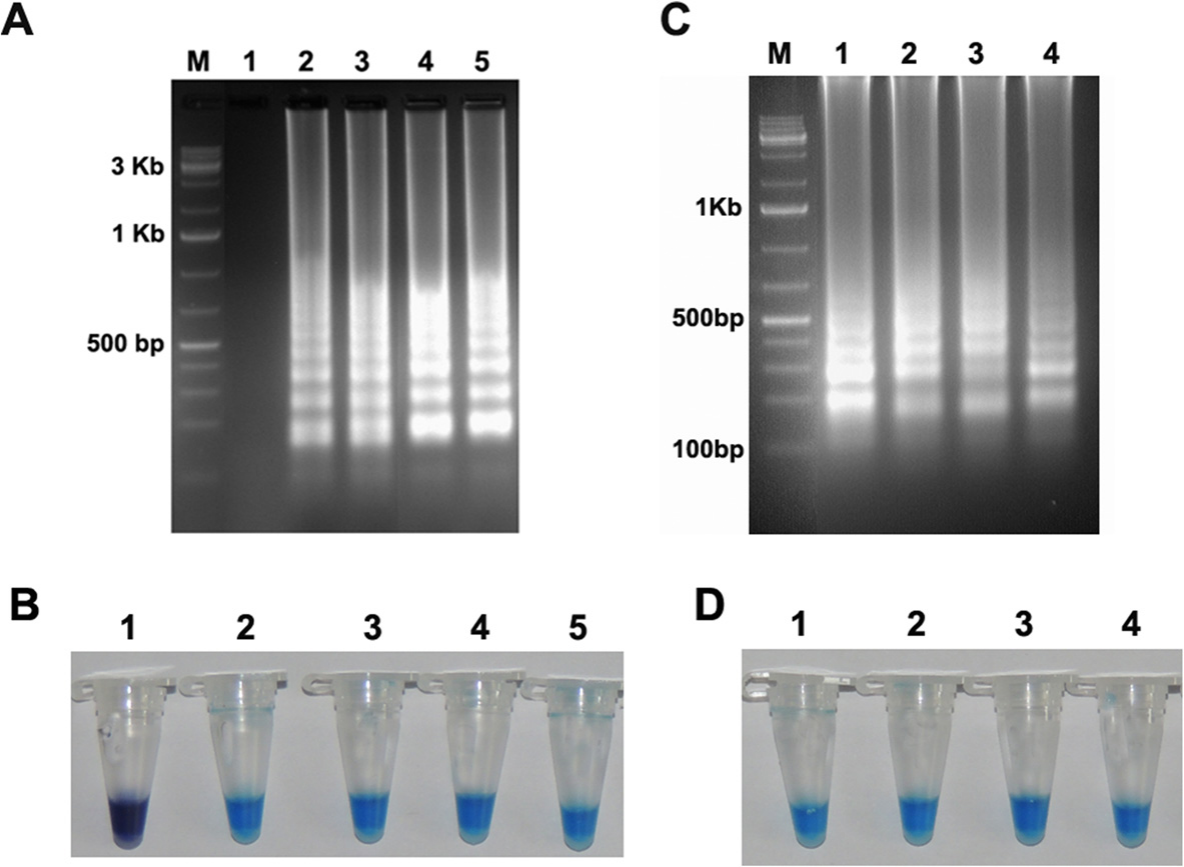
**Optimization of LAMP reaction time and reaction temperature. (A,B)** Optimization of time, **(A)** Assessment was based on 1.5% agarose gel electrophoresis and **(B)** HNB visualization of colour change. Lane 1: 15 min; lane 2: 30 min; lane 3: 45 min; lane 4: 60 min; and lane 5: 90 min. **(C,D)** Optimization of temperature, **(C)** Assessment was based on 1.5% agarose gel electrophoresis and **(D)** HNB visualization of colour change. Lane 1: 63°C; lane 2: 61°C; lane 3: 59°C; and lane 4: 57°C. M indicates a 100 bp DNA marker.

With Foc DNA as the template and the reagents optimized as indicated in the previous section, the optimal LAMP reaction time and temperature were determined. When LAMP was conducted at 63°C, positive results were obtained with times of 30 to 90 min whether assessment was based on gel electrophoresis (Figure 2A) or by HNB-visualization (Figure 2B) but the ladder-like pattern produced by gel electrophoresis was strongest at 60 min. When LAMP was conducted for 60 min with a range of test temperatures, all temperatures produced a positive reaction whether assessment was based on gel electrophoresis (Figure 2C) or by HNB-visualization (Figure 2D) but the bands obtained with gel electrophoresis were most intense at 63°C (Figure 2C). In summary, LAMP of the EF-1alpha gene was optimal when conducted at 63°C for 60 min.

### Evaluation of LAMP assay

All the 50 Foc isolates collected from diverse geographical locations in India and representing various races were tested by LAMP and showed the positive reaction as indicated by a sky-blue colour. The results were consistent with PCR method.

### Specificity of the LAMP assay

**Figure 3:**
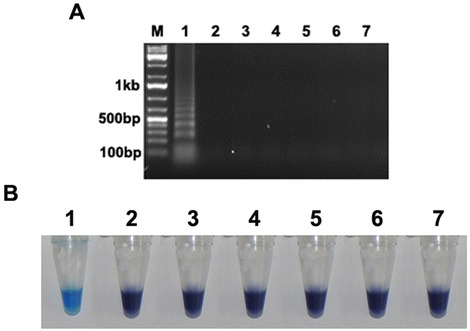
**Specificity of the LAMP assay for** ***Fusarium oxysporum*** **f. sp.** ***ciceris*** **detection. (A)** Detection of *Fusarium oxysporum* by LAMP assay on the basis of 1.5% agarose gel electrophoresis. M indicates 100 bp DNA marker. **(B)** Visual inspection of LAMP reaction tubes. Positive reactions turned sky blue after the addition of HNB. 1: *Fusarium oxysporum* f. sp*. ciceris*, 2: *Fusarium acuminatum*, 3: *Fusarium udum*, 4: *Fusarium solani*, 5: *Rhizoctonia bataticola*, 6: *Alternaria alternata* and 7: *Phytophthora cajani.*

For the LAMP specificity assay, the assay was performed with template fungal DNA from six other fungal cultures (*Fusarium acuminatum, Fusarium udum, Fusarium solani, Rhizoctonia bataticola, Alternaria alternata* and *Phytophthora cajani*) as well as DNA isolated from infected field samples of chickpea (Black root rot caused by *Fusarium solani*, Dry root rot caused by *Rhizoctonia bataticola* and Alternaria blight caused by *Alternaria alternata*). At optimum condition, no positive amplification was observed in case of other fungal DNA samples. Same result was obtained when products were assessed by gel electrophoresis or by HNB-visualization. After incubation at 63°C for 60 min, the LAMP assay was positive only for Foc, i.e., no positive DNA products were observed when other plant pathogenic fungi (Figure 3A,B) were used as template. This was true whether assessments were based on gel electrophoresis (Figure 3A) or HNB visualisation (Figure 3B). Similarly, in case of infected plant samples, DNA isolated from Fusarium wilt infected chickpea plants showed positive reaction (Additional file 1: Figure S1). These results indicated that the LAMP technique developed in this study is highly specific for Foc and has distinguished between Foc and six above mentioned common plant pathogenic fungi.

### Sensitivity of the LAMP assay

**Figure 4:**
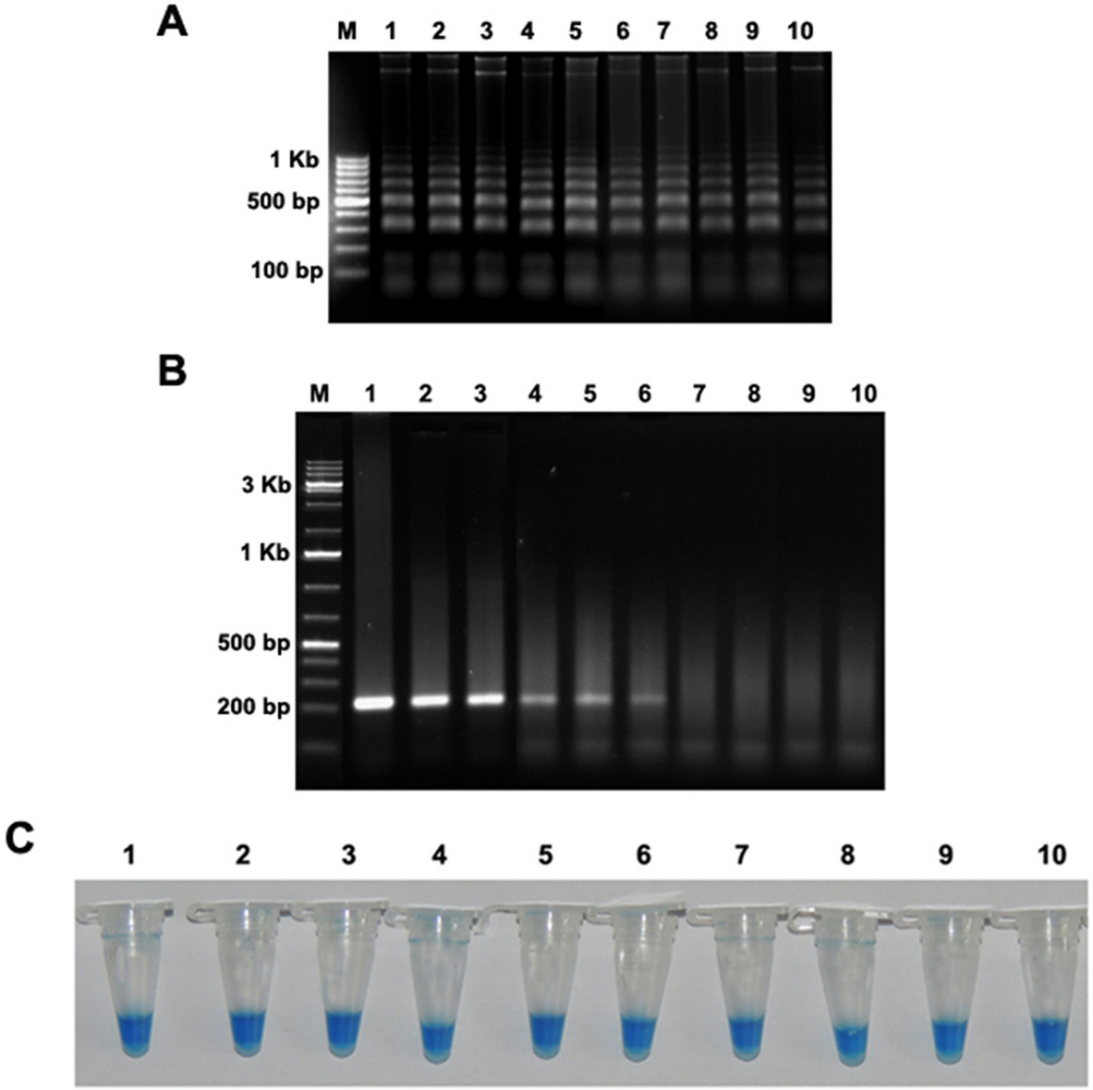
**Comparative sensitivity of LAMP and conventional PCR analysis for detection of** ***Fusarium oxysporum*** **f. sp.** ***ciceris*** **using serial dilutions of DNA as template. A)** Electrophoresis analysis of LAMP. Lane 1: 10 μg; lane 2: 1 μg; lane 3: 100 ng; lane 4: 10 ng; lane 5: 1 ng; lane 6: 100 pg; lane 7: 10 pg; lane 8: 1 pg; lane 9: 100 fg; lane 10: 10 fg. Lane M, 100 bp DNA Marker. **B)** Electrophoresis analysis of conventional PCR with ITS-1 and ITS-4 primers. Lane M, DNA marker (1 Kb). **C)** Visual detection of LAMP assay by addition of HNB.

The sensitivity of the LAMP assay was assessed using serial dilutions of the fungal DNA as template under optimized condition. Figure 4A shows that the LAMP products consisted of ladder like DNA fragments were amplified upto 10 fg, giving a sensitivity of about 100 pg of the fungal DNA. Although the amplified DNA fragments were slightly faint in 10 fg sample than those produced by a greater amount of DNA. In contrast, when same amount of DNA was used in conventional PCR no such amplification was obtained at higher dilutions (Figure 4B). Similar result was obtained when detection was carried out by involving HNB dye also (Figure 4C). It indicated the detection limit of genomic DNA for LAMP assay was 10 fg and 100 pg for conventional PCR. These results also indicated that visual detection can be correlated with the results from agarose gel electrophoresis.

## Discussion

To the best of our knowledge, this is the first report on the application of the LAMP assay for detection of Foc. Relative to conventional PCR, the LAMP assay reported here is easier to perform and more rapid, and the results are easier to evaluate. LAMP operates under isothermal conditions; the optimal temperature for detection of Foc was determined to be 63°C in this study. LAMP is also rapid; for detection of Foc, 60 min was determined to be optimum. Because LAMP is conducted at one temperature, no time is mislaid as a result of changes in temperature, as is the case with thermal cycling with PCR. Moreover, LAMP requires only a regular laboratory bath or heat block that can provide a constant temperature of 63°C. Another very important advantage of LAMP is that the amplified products can be visually detected by adding the dye hydroxynaphthol blue (HNB), i.e., electrophoresis is not required. Because the LAMP assay is simple, it should be useful even for those laboratories and research institutes that are unfamiliar with PCR or other methods of molecular analysis. The simplest way of detecting LAMP products is to inspect the white turbidity that results from magnesium pyrophosphate accumulation, as a by-product of the reaction, by naked eye [26].

However, a small amount of this white precipitate is not always distinguishable from other white precipitates, such as proteins or carbohydrates, derived from the templates. As an alternative method, this study employed metal-ion indicators such as hydroxynaphthol blue (HNB) for low-cost detection of amplified DNA. The results obtained by this system were consistent with those obtained by gel electrophoresis. Since the detection can be accomplished in a closed system without opening the reaction tubes, the risk of contamination is much lower than in gel electrophoresis or by adding dye at the end of the reaction. Theoretically, it should be possible to replace hydroxynaphthol blue (HNB) with other dyes such as SYBR Green I [27-29], Ethidium bromide, EvaGreen [30], and PicoGreen [31] which are reported to be useful for the detection of LAMP products.

The LAMP assay developed here for detection of Foc using six primers: F3, B3, FIP, BIP, LF and LB. To confirm the efficiency and specificity of the six primers, we used DNA extracted from 50 isolates of Foc and from six other plant-pathogenic fungi as templates for LAMP assay. The LAMP assay correctly distinguished between Foc and other pathogens, as experimentation showed its inability to produce an amplification product from other pathogenic genome, thus confirming the LAMP assay and the primers designed here are specific for Foc.

As the LAMP reaction progresses, pyrophosphate ions are produced; these bind to Mg^2+^ ions and form a white precipitate of magnesium pyrophosphate. Therefore, the results of the LAMP can be judged by the unaided eye. This characteristic feature of the LAMP reaction means that the reaction end point can be detected simply by gauging the presence of a precipitate.

The addition of HNB before the LAMP reaction facilitates the determination of a positive result. HNB is a colorimetric indicator of calcium and alkaline earth metal ions. In a LAMP reaction mixture, dNTPs can influence the colour of HNB by the chelating with the Mg^2+^ ions. In the presence of HNB, the colour gradually changes from violet to sky blue as the dNTPs decrease during amplification [20]. In this study, 150 μM HNB successfully distinguished between positive and negative samples. Compared to other methods used to visually detect endpoints, such as those based on the visualization of turbidity [32], the addition of DNA intercalating dyes [33-35], or the use of calcein [17], the use of HNB is simpler [36]. HNB can be added before incubation so that amplification is completed in a closed tube system, and detection of the colour change requires no equipment. The positive and negative reactions obtained with LAMP and HNB were confirmed when the LAMP products were subjected to gel electrophoresis analysis in the current study.

LAMP reaction might be facilitated by the addition of loop-forward and loop backward primers [19]. In the present study, we have identified suitable loop-forward primer and backward primer, and so we used loop-forward primer and backward primer to accelerate the reaction (Table 1). This improved the reaction time and efficiency.

**Table 1:** **Information on the primers used in this study**

**Primer name**	**Sequence (5′- 3′)**	**Length (bp)**
F3	ACAACCTCAATGAGTGCG	18
B3	CATGAGCGACAACATACCA	19
FIP (F1C + F2)	CCAGGCGTACTTGAAGGAACC	41
	GTCAAGCAGTCACTAACCAT	
BIP (B1C + B2)	AGCGTGAGCGTGGTATCAC	37
	ACGGTGACATAGTAGCGA	
LoopF	GCTCAGCGGCTTCCTATT	18
LoopB	CTCTGGAAGTTCGAGCATCC	20
ITS-1	TCCGTAGGTGAACCTGCGG	19
ITS-4	TCCTCCGCTTATTGATATGC	20

To test the LAMP utility, 50 isolates of all Foc were subjected to LAMP and conventional PCR. Compared with the other methods, the newly developed LAMP assay significantly improved the detection efficiency. Therefore, the LAMP assay can be used for detection of Foc in plants.

## Conclusion

In conclusion, it was found that a LAMP assay combined with HNB is simple, rapid, sensitive and specific. Because this LAMP assay does not require specialized equipment, it can be used in the field for the rapid detection of Foc. This is the first report of the use of LAMP assay for the detection of Foc. It is a promising assay for extensive application and rapid diagnosis of Foc infection in the laboratory and will be very useful for monitoring the disease complex in the field further suggesting management strategies.

## Methods

### Fungal culture

A total of 50 Foc isolates collected from 21 locations representing 12 states and five agro-ecological zones of India (Central zone, North east plain zone, North hill zone, North west plain zone and South zone) were used in the present study to validate the LAMP assay (Table 2). All the isolates were purified and mono-conidial cultures were maintained on potato dextrose agar (PDA) slants at 4°C. The pathogenicity of all the isolates was proved following root-dip inoculation method under controlled environmental conditions [37]. Other Plant Pathogens used in this study are maintained in a collection in the Legumes Pathology Division, ICRISAT, India.

**Table 2:** **Passport information of isolates used in** ***Fusarium oxysporum*** **f. sp.** ***ciceris*** **LAMP assay development and their LAMP reaction**

**S. no**	**Isolate ID**	**Location**	**State**	Agro-ecological zone*	**Latitude**	**Longitude**	**Elevation (m)**	**LAMP reaction**
1	Foc_1	Patancheru	Andhra Pradesh	SZ	17°31'53" N	78°15'54" E	516	+
2	Foc_2	Patancheru	Andhra Pradesh	SZ	17°31'53" N	78°15'54" E	516	+
3	Foc_3	Patancheru	Andhra Pradesh	SZ	17°31'53" N	78°15'54" E	516	+
4	Foc_4	Patancheru	Andhra Pradesh	SZ	17°31'53" N	78°15'54" E	516	+
5	Foc_5	Patancheru	Andhra Pradesh	SZ	17°31'53" N	78°15'54" E	516	+
6	Foc_6	Patancheru	Andhra Pradesh	SZ	17°31'53" N	78°15'54" E	516	+
7	Foc_7	Hisar	Haryana	NW PZ	29°10'00" N	75°43'00" E	202	+
8	Foc_8	Hisar	Haryana	NWPZ	29°10'00" N	75°43'00" E	202	+
9	Foc_9	Hisar	Haryana	NWPZ	29°10'00" N	75°43'00" E	202	+
10	Foc_11	Dhaulakuan	Himachal Pradesh	NHZ	30°28'00" N	77°05'00" E	468	+
11	Foc_12	Gulbarga	Karnataka	SZ	17°19'59" N	76°49'59" E	458	+
12	Foc_13	Junagadh	Gujarat	CZ	21°31'00" N	70°28'00" E	119	+
13	Foc_14	Junagadh	Gujarat	CZ	21°31'00" N	70°28'00" E	119	+
14	Foc_16	Badnapur	Maharashtra	CZ	19°52'00" N	75°43'60" E	498	+
15	Foc_17	Badnapur	Maharashtra	CZ	19°52'00" N	75°43'60" E	498	+
16	Foc_20	Delhi	Delhi	NWPZ	28°40'00" N	77°13'00" E	213	+
17	Foc_21	Ludhiana	Punjab	NWPZ	30°54'00" N	75°51'00" E	243	+
18	Foc_22	Ludhiana	Punjab	NWPZ	30°54'00" N	75°51'00" E	243	+
19	Foc_23	Gurdaspur	Punjab	NWPZ	32°03'00" N	75°27'00" E	241	+
20	Foc_25	Kanpur	Uttar Pradesh	NEPZ	26°28'00" N	80°21'00" E	128	+
21	Foc_26	Kanpur	Uttar Pradesh	NEPZ	26°28'00" N	80°21'00" E	128	+
22	Foc_28	Pantnagar	Uttarakhand	NWPZ	29°20'04" N	79°28'25"E	344	+
23	Foc_29	Kurnool	Andhra Pradesh	SZ	15°48'00" N	78°04'00" E	289	+
24	Foc_31	Akola	Maharashtra	CZ	20°43'59" N	77°00'00" E	283	+
25	Foc_33	Jabalpur	Madhya Pradesh	CZ	23°10'01" N	79°57'00" E	403	+
26	Foc_34	Jabalpur	Madhya Pradesh	CZ	23°10'01" N	79°57'00" E	403	+
27	Foc_36	Patancheru	Andhra Pradesh	SZ	17°31'53" N	78°15'54" E	516	+
28	Foc_37	Dharwad	Karnataka	SZ	15°28'00" N	75°01'00" E	700	+
29	Foc_38	Patancheru	Andhra Pradesh	SZ	17°31'53" N	78°15'54" E	516	+
30	Foc_39	Kanpur	Uttar Pradesh	NEPZ	26°28'00" N	80°21'00" E	128	+
31	Foc_45	Delhi	Delhi	NWPZ	28°40'00" N	77°13'00" E	213	+
32	Foc_47	Dharwad	Karnataka	SZ	15°28'00" N	75°01'00" E	700	+
33	Foc_51	Dhaulakuan	Himachal Pradesh	NHZ	30°28'00" N	77°05'00" E	468	+
34	Foc_65	Patancheru	Andhra Pradesh	SZ	17°31'53" N	78°15'54" E	516	+
35	Foc_76	Satna	Madhya Pradesh	CZ	24°34'59" N	80°49'59" E	318	+
36	Foc_79	Rewa	Madhya Pradesh	CZ	24°31'59" N	81°18'00" E	275	+
37	Foc_87	Rajnandgaon	Chhattisgarh	CZ	21°06'00" N	81°02'00" E	330	+
38	Foc_92	Sehore	Madhya Pradesh	CZ	23°12'00" N	77°04'59" E	502	+
39	Foc_93	Patancheru	Andhra Pradesh	SZ	17°31'53" N	78°15'54" E	516	+
40	Foc_95	Kanpur	Uttar Pradesh	NEPZ	26°28'00" N	80°21'00" E	128	+
41	Foc_96	Kanpur	Uttar Pradesh	NEPZ	26°28'00" N	80°21'00" E	128	+
42	Foc_100	Jabalpur	Madhya Pradesh	CZ	23°10'01" N	79°57'00" E	403	+
43	Foc_101	Jabalpur	Madhya Pradesh	CZ	23°10'01" N	79°57'00" E	403	+
44	Foc_115	Satana	Madhya Pradesh	CZ	24°34'59" N	80°49'59" E	318	+
45	Foc_116	Satana	Madhya Pradesh	CZ	24°34'59" N	80°49'59" E	318	+
46	Foc_118	Satana	Madhya Pradesh	CZ	24°34'59" N	80°49'59" E	318	+
47	Foc_119	Damoh	Madhya Pradesh	CZ	23°49'59" N	79°27'00" E	354	+
48	Foc_131	Rewa	Madhya Pradesh	CZ	24°31'59" N	81°18'00" E	275	+
49	Foc_132	Satana	Madhya Pradesh	CZ	24°34'59" N	80°49'59" E	318	+
50	Foc_145	Katni	Madhya Pradesh	CZ	23°47'00" N	80° 27'00" E	392	+

*CZ - Central zone, NEPZ - North east plain zone, NHZ - North hill zone, NWPZ - North west plain zone and SZ - South zone.

### DNA extraction

Fungal DNA extraction was done by following cetyltrimethylammonium bromide (CTAB) method [38]. All the 50 isolates were grown in PDB and incubated in a rotary shaker at 120 rpm at 25 ± 1°C for five days. In brief, mycelia were harvested by filtering through mira cloth, and washed repeatedly with sterile distilled water to remove excess of salts adhering to it. One gram mycelium was crushed in liquid nitrogen and transferred into 7.5 ml pre-warmed (65°C) DNA extraction buffer [1 M Tris–HCl (pH 8.0), 5 M NaCl, 0.5 M ethylene diamine tetra acetic acid (EDTA; pH 8.0) and 2% CTAB], mixed well and incubated in a water bath at 65°C with gentle shaking for 45 min. Equal volume of chloroform: isoamyl alcohol (24:1 v/v) was added and mixed gently to denature proteins and centrifuged at 12,857 g for 10 min. DNA was precipitated with 0.6 volume of chilled ethanol and 0.1 volume of 3 M sodium acetate (pH 5.2) and centrifuged at 18,514 g for 15 min. The pellets were washed twice with chilled 70% ethanol, dried at room temperature, re suspended in 100 μl sterile TE (10 mM Tris–HCl buffer and 1 mM EDTA; pH 8) and stored at −20°C. Isolated DNA was run in 0.8% agarose gels and spectrophotometric analysis (Nanodrop spectrophotometer, Thermo Scientific, USA) to check the quality and quantity of DNA. Similarly, genomic DNA from the infected plants from chickpea fields was extracted using PureLink plant total DNA purification kit (Invitrogen, USA) following manufacturer’s instructions. Quality and quantity of DNA was evaluated on 0.8% agarose gel as well as by spectrophotometric analysis and stored at −20°C for further use.

### Primer design

**Figure 5:**
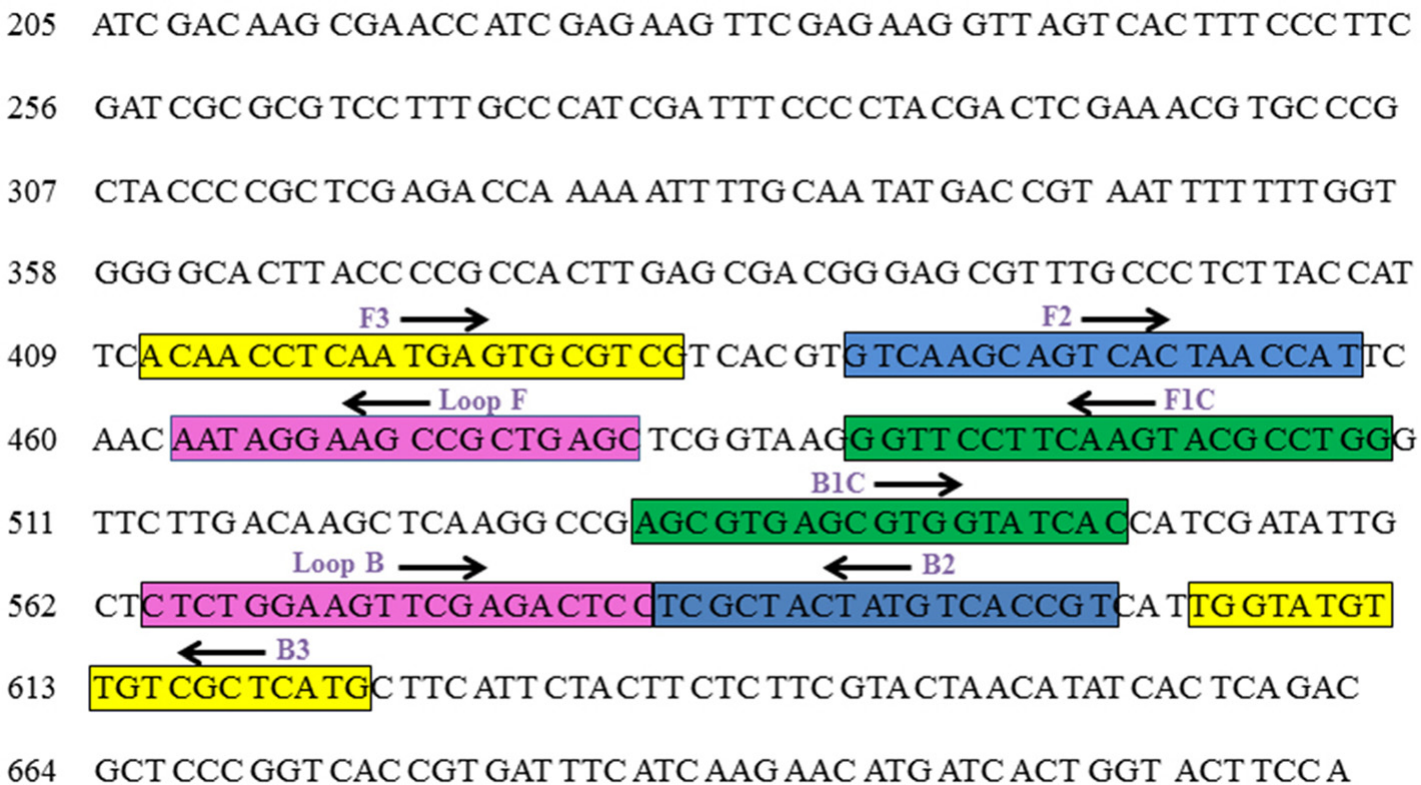
**Schematic representation of Position and sequence of primer sets within the nucleotide sequence of the elongation factor 1 alpha gene of** ***Fusarium oxysporum*** **f. sp.** ***ciceris*** **used for LAMP assay.** Arrows and box indicate the position of the target sequences.

Six specific LAMP primers were designed based on the Foc elongation factor 1 alpha (EF-1alpha) gene (FJ538243). Specific primers based on the EF-1alpha gene sequence alignment were designed for LAMP detection of Foc using the LAMP designer software program (http://lamp-designer.software.informer.com/). The structure of the LAMP primers and their complementarity to target DNA used in this study are shown in Figure 5. A forward inner primer (FIP) consisted of the complementary sequence of F1 (F1C) and F2, and a backward inner primer (BIP) consisted of B1C and B2. The outer primers F3 and B3 are required for initiation of the LAMP reaction. Primer pair ITS-1 and ITS-4 were used for conventional PCR. Information regarding the primer names and sequences is provided in Table 1.

### Optimization of LAMP reaction conditions

The LAMP reaction mixtures (HNB and with or without Foc DNA as template) were incubated for 60 min from 57°C to 63°C to determine the optimal reaction temperature. Then, the LAMP was performed at the optimal reaction temperature for 15, 30, 45, 60, and 90 min to determine the optimal reaction time. The reactions were terminated by heat inactivation at 80°C for 10 min. The assay was assessed based on HNB-visualized colour change and then on gel electrophoresis as described in the previous section.

### Evaluation of the LAMP assay

Total 50 isolates of Foc collected from diverse geographical locations and representing Foc races in India were subjected to LAMP and conventional PCR. When LAMP reactions were finished, they were assessed on colour change and based on gel electrophoresis as described earlier.

### Specificity of the LAMP

LAMP specificity was determined by performing the assay with fungal DNA from Foc and six other plant-pathogenic fungi (*Fusarium acuminatum*, *Fusarium udum*, *Fusarium solani*, *Rhizoctonia bataticola*, *Alternaria alternata* and *Phytophthora cajani*). Similarly LAMP assay was performed with DNA isolated from infected field samples of chickpea (Black root rot caused by *Fusarium solani*, Dry root rot caused by *Rhizoctonia bataticola* and Alternaria blight caused by *Alternaria alternata*) as described earlier at 63°C for 60 min. The assay was assessed based on HNB-visualized colour change and then by gel electrophoresis. Each fungal sample was represented by three replications, and the experiment was performed three times.

### LAMP sensitivity

LAMP sensitivity assay was detected by comparing the assay with conventional PCR method by using different concentrations of DNA sample. The LAMP assay was performed by using a serially diluted DNA samples at concentrations range from 10 μg to 10 fg. The purified DNA was dissolved in double-distilled water, and 1 μl of the solution was used as the template for LAMP. For the conventional PCR run the DNA was amplified with ITS-1 and ITS-4 primers with similar concentrations of DNA. Template DNA from Foc was prepared as described earlier and was serially diluted from 10 μg to 10 fg. The samples were then subjected to LAMP (with HNB) and PCR assays. After completion of the reaction both the reactions were assessed; the LAMP products were visualized as described earlier, while the PCR products were observed by gel electrophoresis.

## Additional file

Additional file 1: Figure S1: Specificity of LAMP assay with DNA isolated from infected chickpea plant. Tube 1: DNA isolated from infected sample of Fusarium wilt showing positive result. Tube 2: DNA isolated from infected sample of Black root rot. Tube 3: DNA isolated from infected sample of Dry root rot, Tube 4: DNA isolated from infected sample of Alternaria blight. Assessment based on HNB visualization of colour change.

## Abbreviations

EF-1alpha: Elongation factor 1 alpha
Foc: *Fusarium oxysporum* f. sp. *ciceris*
HNB: Hydroxynaphthol blue
ITS: Internal transcribed spacer
LAMP: Loop-mediated isothermal amplification
