# Supplementary figures and images for: Development of Loop-Mediated Isothermal Amplification (LAMP) assay for rapid detection of Fusarium oxysporum f. sp. ciceris - wilt pathogen of chickpea
Source: BMC Res Notes. 2015 Feb 11;8:40. doi: 10.1186/s13104-015-0997-z (PMC4332723; doi:10.1186/s13104-015-0997-z)

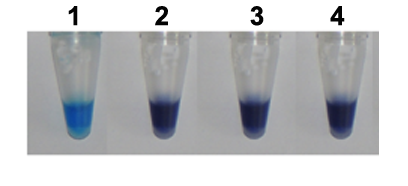

Supplement: Additional file 1: Figure S1: — Specificity of LAMP assay with DNA isolated from infected chickpea plant. Tube 1: DNA isolated from infected sample of Fusarium wilt showing positive result. Tube 2: DNA isolated from infected sample of Black root rot. Tube 3: DNA isolated from infected sample of Dry root rot, Tube 4: DNA isolated from infected sample of Alternaria blight. Assessment based on HNB visualization of colour change. [file 13104_2015_997_MOESM1_ESM.tiff]
